# Hospital Expenditure—The Commissariat

**Published:** 1896-11-21

**Authors:** 


### The Institutional Workshop.

### HOSPITAL EXPENDITURE—THE COMMISSARIAT.

### XXXII.—THE SALE OF WASTE MATERIAL.

The old system of perquisites is now very generally discredited, although, like many other bad old customs, it still lingers in regions where the modern spirit of reform has failed to penetrate. By that system servants obtained a small amount only of their wages in money, and a substantial, if somewhat fluctuating, income derived from tips, subsidies from tradesmen, and the sale of all the waste materials they were thrifty enough to scrape together. If the housemaid were most in the ways of tips, to the cook fell all the more important emoluments resulting from her influence over the supplies. To her also would fall the dripping and miscellaneous gleanings, known as "wash," while the kitchenmaid would rejoice in the bottles and bones, and the upstairs helpers would assiduously collect the rags and waste paper. It was common to contend that by these means all waste was prevented. But the contrary began, when more insight was gained into the working of large establishments, to be convincingly evident. The honesty of even upright persons can hardly fail to be undermined by a system under which all extravagance in the providing or use of common necessaries directly benefits their own pockets. Honesty is, in fact, very largely a matter of tradition and circumstance, and in this matter of perquisites the conscientious became discouraged, the weak became corrupted, and the dishonest plundered far and wide. The Christmas present from butcher or baker, originally a mere token of goodwill and gratitude, tended to degenerate into a painful exaction, and is still very commonly so levied under threats by unscrupulous servants as hardly to be distinguishable from blackmail. The wastefulness provoked by allowing members of the household to sell any article out of the institution worked at length the downfall of the custom. It is now in all well-regulated houses practically dead, and where it is still tacitly connived at is but one among symptoms of general slackness.

There is no doubt that this source of revenue, now closed to the servants, whose wages have in consequence in many institutions undergone a considerable rise, is one not to be despised. It appears in some hospital reports among receipts to a figure of some dignity, and is wholly absent from that of others, owing possibly to the fact of the waste material being accepted by one of the tradesmen as a set-off to goods supplied. Needless to say, whatever arrangement may be entered into to pay in kind, all receipts from this source should duly appear on the credit side, and all goods provided be charged at their purchasable value on the other. One important exception should be made in the case of the return of beer or mineral-water bottles, which should more properly be credited on the bill of the purveyor of these articles. They have, in fact, never been actually bought, but rather lent, and to include the cost of bottles meant to be returned in the cost of malt liquor or mineral waters would be to convey an entirely erroneous impression as to the amount of these articles consumed in the hospital.

The following are the sums credited to the account of some of the London and provincial hospitals for sale of kitchen stuff, waste materials, &c., in 1893:—

| Daily average of inmates. | | £ |
|---|---|---|
| 733 | London Hospital (waste materials) | £122 |
| — | King's (including bottles, £56; old stores, £8) | 116 |
| 242 | Charing Cross | 90 |
| 240 | University | 87 |
| 373 | St. Mary's (old materials) | 60 |
| 506 | St. George's | 49 |
| 182 | Royal Free | 49 |
| 97 | Metropolitan (waste stores) | 47 |
| 106 | Great Northern Central | 32 |
| 283 | Seamen's | 31 |
| 224 | Westminster | 4 |
| 356 | Birmingham General (including bottles, £56) | 89 |
| 256 | Sheffield General | 63 |
| 367 | Newcastle-on-Tyne | 54 |
| 514 | Leeds General | 48 |
| 223 | Hull Royal | 43 |
| 262 | Bristol General | 40 |
| 143 | Derbyshire Royal | 28 |
| 145 | York County | 24 |
| 320 | Liverpool Royal | 21 |
| 133 | Halifax | 18 |
| 174 | Oxford Radcliffe | 16 |
| 209 | Sussex County | 15 |
| 198 | Norfolk and Norwich | 7 |
| 779 | Glasgow Royal (old materials) | 129 |
| 553 | Glasgow Western (refuse) | 108 |
| 171 | Belfast Royal (refuse) | 16 |

Having regard to the wide diversity of these receipts, it is natural to inquire what causes may give rise to this diversity, and what sums it would be reasonable to expect from this curiously ill-defined source of income. Taking the items one by one, the first and most important is dripping.

Dripping is an article of some value. A ready sale can be obtained for it in any locality at from 4d. to 8d. per pound, and as about ten per cent. of all the meat cooked resolves itself into this form, the weekly yield should be accurately ascertained and thriftily dealt with. Not much dripping can be used in the hospital dietary. For the patients it is, of course, out of the question, and nurses are not found to favour it in any form, except that of an occasional cake or pie-crust. Some servants are glad to use it for breakfast or tea in place of butter, but not very commonly in London. Much is often sold at a reduced price of 3½d. or 4d. per pound to scrubbers and laundry hands, and some hospitals situated in exceptionally poor neighbourhoods give away a good deal of dripping and other "broken meats" among maternity patients and others who have been under treatment. One or two hospitals again, fortunate in the possession of their own convalescent homes, find the dripping in great request among the hungry convalescents. In all these cases full value is obtained for this article, although it does not figure in the receipts. The housekeeper should, however, bear in mind what reduction she has a right to expect in the amount used of butter, lard, and suet when dripping takes the place of any one of these. She should require from the cook the weekly weight of dripping, and carefully check the sale, whether to outside helpers or dealers. In hospitals where not much dripping is used, and none given away, it would be reasonable to expect a weekly profit from this source of from 10s. to 12s. 6d. for every hundred persons boarded, reckoning that in such an establishment at least 30 lbs. of dripping would be produced in the week.

Bones are not a very lucrative article; still, at 6 lbs. for 1d. a sum of from 17s. 6d. to £1 1s. a year may be easily obtained from them in a household of this size.

More important is the item known variously as "wash" or "swill," for which proper receptacles should invariably be provided. This consists of parings of vegetables, leavings from the plates, sour milk or beef-tea, stale crusts, &c., &c., and is of considerable value in the feeding of pigs and poultry. Its market price varies in different localities, but may be estimated at from 2s. 6d. to 5s. a week for every 100 persons.

Waste paper is worth a good deal less now than formerly. Still, the weight of printed and written matter showered down on the secretarial department in any large institution may with very little trouble be turned to account, and stand at the end of the year for a new guinea subscriber.

Other waste materials are of a less constant nature. The refurnishing of any department entails the sale of the worn-out articles, and in all but the newest institutions a certain amount of renewing goes on year by year. Included among these rummage articles are also those well-intentioned cases of ancient accumulations, adapted to no purpose but that of the second-hand dealer, which every hospital is liable to receive from its well wishers.

Omitting all such occasional items, it is plain, as a matter not of theory but of actual experience, that every institution may derive from its own waste products a sum, either in money or money's worth, of from £30 to £50 for every 100 inmates boarded, and this sum is surely well worth the collecting, even through the undeniably humble medium of "swill and drip."

